# Micron-scale phenomena observed in a turbulent laser-produced plasma

**DOI:** 10.1038/s41467-021-22891-w

**Published:** 2021-05-11

**Authors:** G. Rigon, B. Albertazzi, T. Pikuz, P. Mabey, V. Bouffetier, N. Ozaki, T. Vinci, F. Barbato, E. Falize, Y. Inubushi, N. Kamimura, K. Katagiri, S. Makarov, M. J.-E. Manuel, K. Miyanishi, S. Pikuz, O. Poujade, K. Sueda, T. Togashi, Y. Umeda, M. Yabashi, T. Yabuuchi, G. Gregori, R. Kodama, A. Casner, M. Koenig

**Affiliations:** 1 LULI, CNRS, CEA, École Polytechnique, UPMC, Univ Paris 06: Sorbonne Universités, Institut Polytechnique de Paris, F-91128 Palaiseau cedex, France; 2 Institute for Open and Transdisciplinary Research Initiative, Osaka University, Osaka, Japan; 3 Joint Institute for High Temperatures RAS, Moscow, Russia; 4 Université de Bordeaux-CNRS-CEA, CELIA, UMR 5107, Talence, France; 5 Graduate School of Engineering, Osaka University, Osaka, Japan; 6 Institute of Laser Engineering, Osaka University, Suita, Osaka Japan; 7 CEA-DAM, DIF, Arpajon, France; 8 Japan Synchrotron Radiation Research Institute, Hyogo, Japan; 9 RIKEN SPring-8 Center, Hyogo, Japan; 10 Department of Physics of accelerators and radiation medicine, Faculty of Physics, Lomonosov Moscow State University, Moscow, Russia; 11 General Atomics, Inertial Fusion Technologies, San Diego, CA USA; 12 National Research Nuclear University ‘MEPhi’, Moscow, Russia; 13 Université Paris-Saclay, CEA, LMCE, Bruyères-le-Châtel, France; 14 Institute for Planetary Materials, Okayama University, Tottori, Japan; 15 Department of Physics, University of Oxford, Oxford OX1 3PU, United Kingdom; 16 CEA-CESTA, 15 avenue des Sablières, CS 60001, 33116 Le Barp Cedex, France

**Keywords:** Fluid dynamics, Laser-produced plasmas, Imaging techniques

## Abstract

Turbulence is ubiquitous in the universe and in fluid dynamics. It influences a wide range of high energy density systems, from inertial confinement fusion to astrophysical-object evolution. Understanding this phenomenon is crucial, however, due to limitations in experimental and numerical methods in plasma systems, a complete description of the turbulent spectrum is still lacking. Here, we present the measurement of a turbulent spectrum down to micron scale in a laser-plasma experiment. We use an experimental platform, which couples a high power optical laser, an x-ray free-electron laser and a lithium fluoride crystal, to study the dynamics of a plasma flow with micrometric resolution (~1μm) over a large field of view (>1 mm^2^). After the evolution of a Rayleigh–Taylor unstable system, we obtain spectra, which are overall consistent with existing turbulent theory, but present unexpected features. This work paves the way towards a better understanding of numerous systems, as it allows the direct comparison of experimental results, theory and numerical simulations.

## Abstract (web summary)

Turbulence effects explored use macroscale systems in general. Here the authors generate a turbulent plasma using laser irradiation of a solid target and study the dynamics of the plasma flow at the micron-scale by using scattering of an XFEL beam.

## Introduction

**Fig. 1:**
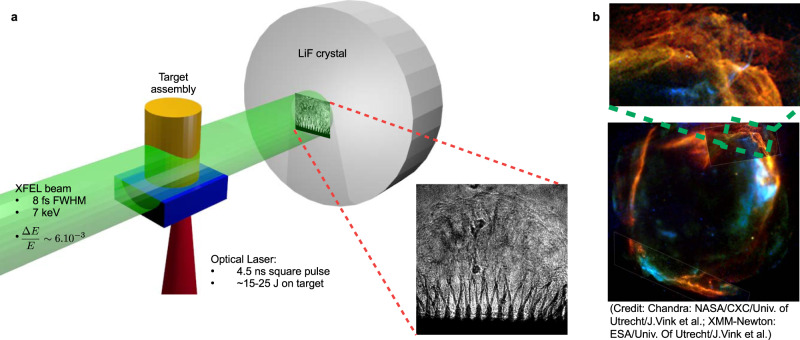
Experimental setup. Diagram of the overall experiment setup (**a**) and Chandra’s observation of RCW 86 (**b**). The plasma expansion inside the laser-driven target is diagnosed using a XFEL beam (>1 mm diameter, 8 fs, centred on a 7 keV emission with a Δ*E*/*E* = 6 × 10^−3^) and lithium fluorine crystal. This expansion is Rayleigh–Taylor unstable, as in the supernovae remnant.

Hydrodynamic turbulence occurs in a variety of systems as a direct consequence of the non-linearity of the fluid equations^1–4^. In high-energy-density physics (HEDP), it permeates every scale from inertial confinement fusion^5–9^ to astrophysical-object evolution^10–12^. This chaotic phenomenon is believed to develop when the viscosity of a flow is negligible, which is often considered to be the case in plasma physics (especially in HEDP). It leads to the creation of 3D eddies of decreasing size, which, carry energy in a cascade from the large scale of injection, to smaller ones, where dissipation occurs. These eddies, whose distribution becomes isotropic at a small enough spatial scale, lead to a global homogenisation of the fluid. This mixing phenomenon plays an important role in numerous systems, either in the laboratory or in the Universe. In inertial confinement fusion, it can hinder, or even prevent, ignition. In astrophysics, turbulence is also believed to strongly influence supernovae explosions^12^ and can be found in their remnants (see Fig. 1a), as well as in the local non-homogeneous aspect of the interstellar medium, thus affecting the star formation process^13–15^ and the propagation of cosmic rays^16,17^. Although multiple studies have already been performed, including HEDP^3,18^, our understanding of turbulence, even in the classical hydrodynamic case, remains incomplete^19^.

The study of turbulent plasma flows includes further complexity (magnetic fields, multiples fluids...), which deviates from the standard approach. Thus, the classical energy cascade and dissipation microscales of the Kolmogorov theory^1^ may not be fully applicable when dealing with plasma^20^. Usually, turbulence theories are investigated through both numerical simulations and experiments, each with their own limitations. Many experiments studying turbulence in HEDP are restricted to macroscale metrics, such as the interface mix-width, since diagnostics can not resolve higher-order features^21^. This constraint severely limits the utility of the experimental approach, since the smallest spatial scales are often non-negligible and are a subject of interest in turbulence studies. For these scales, simulations are often required. Yet, controlled and well-diagnosed experiments are nevertheless mandatory to constrain the predictions of different theories and simulations.

Here, we show results from an experiment where a laser-produced Rayleigh–Taylor unstable plasma flow evolves towards a possibly turbulent state. The main diagnostic, an x-ray radiography platform, coupling an x-ray free-electron laser (XFEL) and a lithium-fluoride crystal (LiF)^22,23^, allowed us to take time-resolved images with sub-micron spatial resolution over a large field of view (>1 mm^2^ corresponding to the XFEL beam size). From the obtained radiographs, we extract intensity spatial spectra, which can be associated to velocity spectra. These spectra are compared to turbulence theories and unaccounted features are discussed. To complete this study, we perform matching simulations using the FLASH4 code^24,25^. They have been used to infer experimentally unmeasured parameters such as the pressure or the fluid velocity, which are necessary for our analysis.

## Results

### Experimental setup and diagnostics

The experiment was conducted at the SACLA facility in Japan^26,27^, with the experimental setup displayed in Fig. 1. A high-power laser beam was used to drive multi-layer targets, whose design was tested in previous experiments^28^ (See Methods for further details). They allow the expansion and deceleration of a plasma into a low-density foam, leading to the development of Richtmyer–Meshkov^29,30^ and Rayleigh–Taylor^31–34^ instabilities (RTI) that evolve into turbulence. The inclusion of a modulation on the surface of the pusher layer favours the growth of a given Fourier mode via the instability, which allows precise control of our experimental conditions. Both mono-mode (40 μm wavelength) and bi-mode modulations (15 and 40 μm wavelengths) were used as variations for the initial conditions.

**Fig. 2:**
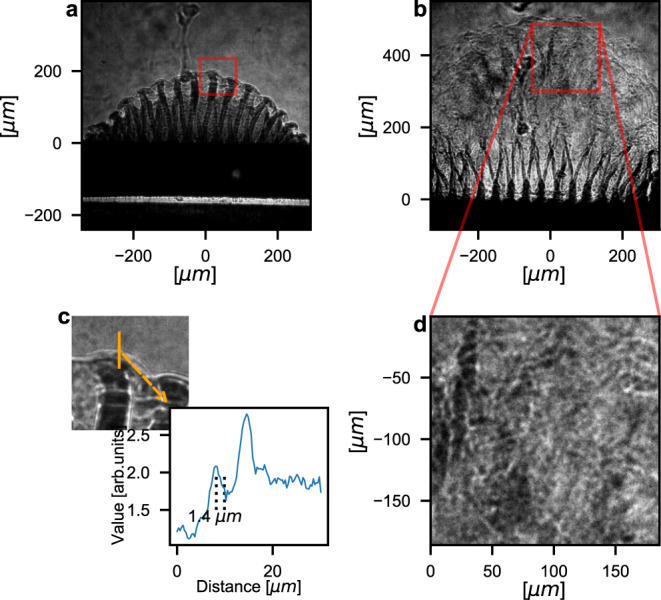
Experimental radiographs of the mono-mode targets. Experimental radiographs of mono-mode targets obtained before (40 ns (**a**) and (**c**)) and after (60 ns (**b**) and (**d**)) the transition to turbulence between 50 and 60 ns. Following the initial Rayleigh–Taylor growth and pusher expansion (curved modulated surface of the two first radiograph), the system becomes turbulent. The structure becomes blurry and the power spectrum nearly isotropic, as can be observed on the zoom of the 60 ns radiograph. A precise study was made possible thanks to the high resolution of the radiography, which is illustrated on the zoom and line-out of the 40 ns radiograph (**c**).

The unstable interface between the pusher and foam was examined using a short-pulse (<10 fs^35^), nearly mono-chromatic (7 keV), collimated XFEL beam combined with a LiF crystal, used as a detector. The resulting x-ray radiographs^36^ present cutting-edge high-resolution (see Fig. S3 in the Supplementary Information). It should be note that this is a successful utilisation of this diagnostic on a plasma in motion. This radiography configuration enables a sub-micron resolution of ~0.6 μm, taking into account the secondary electron avalanche inside the detector^37,38^. The actual experimental resolution was ~1.5 μm due to the presence of phase-contrast (PC) effects^39^. These effects are due to the propagation of the highly coherent beam after its propagation through the target (see line-out of Fig. 2c). This process enhances the contrast of the radiography, as it then depends on the phase in addition to the usual absorption.

The resulting images depend on the projected complex optical path length, the integral of the optical index along the x-ray path, through the plasma, and hence on the material composition and density. To enhance the contrast of the radiographs, the expanding layer of the target, the pusher, was doped with bromine. This element possesses a higher atomic number than the plastic, which composes the major part of the target. Consequently, it has a higher opacity at the XFEL beam energy. At early times, the good contrast is a result of the difference in density between the pusher and the foam. Whereas later in time, when the density tends to become uniform, the bromine ensures the observed contrast. In this way, we obtain high-contrast images throughout the entire experiment.

### Plasma evolution

The evolution of the expanding plasma was measured up to 80 ns after the beginning of the main laser pulse. In Fig. 2, two radiographs, characteristic of a mono-mode target, are shown (a, b). The curvature present in the data is due to the finite laser focal spot size (250 μm). The spike and bubble evolution are clearly observed (a,c), and the RTI growth can be studied up to ~50 ns (see Fig. S2 of the Supplementary Information). The high resolution of our diagnostic allows the investigation of the finest morphological details of the RTI spike extremities (see Fig. 2c). This represents a new horizon in the validation of RTI simulations, which, depending on the models employed, predict different spike morphologies^40^. At later times, the expanding plasma appears as streaked and chaotic; the RTI structures are lost (see Fig. 2b and d). The loss of the initial conditions and the resulting isotropy is consistent with a turbulent flow. The transition between these two phases occurs between 50 ns and 60 ns for the mono-mode targets.

**Fig. 3:**
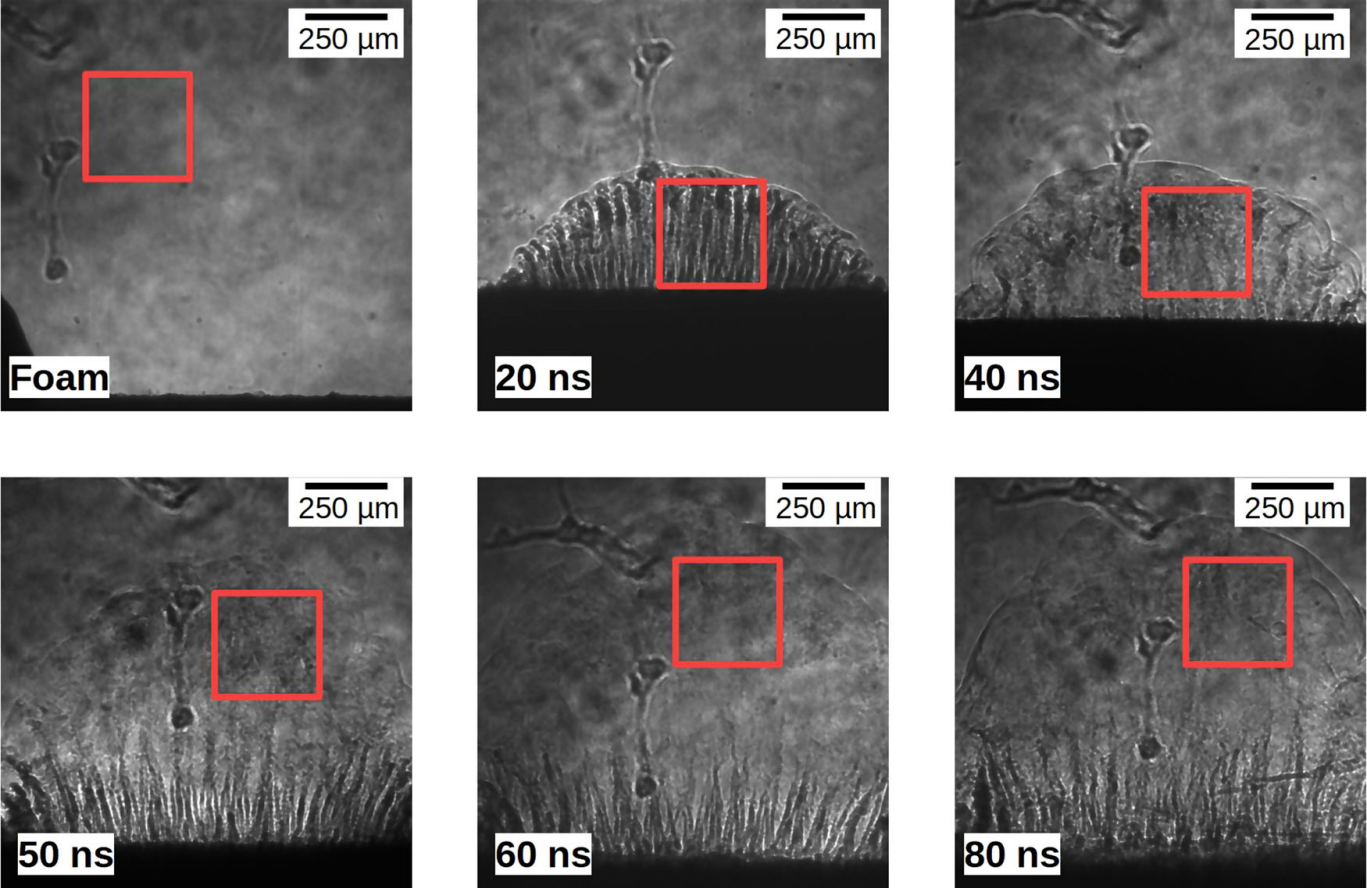
Experimental radiograph showing the dynamic of the bi-mode targets. The position of the ROI taken to calculate the spectrum of Fig. 4 is shown with a red square.

As can be seen in the dynamics shown in Fig. 3, the transition happens at earlier times for bi-mode targets (around 40 ns). This difference might be due to the enhancement of non-linear effects and mode coupling of a multi-wavelength perturbations^41^. This phenomenon is highlighted on the 20 ns radiograph. The more pronounced non-linear effects can result in a quicker destabilisation of the flow, leading more rapidly to the turbulence, since the energy injection happens at multiple wavelengths.

### Simulations and dimensionless numbers

To ascertain the nature of the flow at late time, it is necessary to study not only its spectrum, but also the actual plasma regime. One of the characteristics of a turbulent flow is a high Reynolds number (Re) at the injection scale, for instance higher than 1.5 × 10^6^ according to P.E. Dimotakis^42^. To calculate this number, the characteristic length (*L*), velocity (*u*) and viscosity (*ν*) of the flow are mandatory. Except for *L*, these parameters can not be obtained from the radiography and so simulations were performed to determine their value at the beginning of the turbulent phase.

The 2D hydrodynamic simulations, performed with the FLASH4 code, globally reproduce the evolution of our plasma flow up to the beginning of the turbulent phase. In particular, the morphology of the interface, its position and the RTI growth are correctly reproduced up to 50 ns (see Figs. S1 and S2 of the Supplementary). However, the observed blurry aspect is not recreated in simulations, which make them inappropriate for the detailed study of the turbulent-like phase. This might be due to the coarse resolution or to the absence of direct dissipation mechanism (Euler equations). Indeed, the simulations are limited in resolution and do not reach the dissipation scale, thus limiting the effective Reynolds number of the simulations (Re = (*L*/*η*)^4/3^, see the following discussion or method for limitations). The absence of the third dimension in the simulations could also contribute (see Section II of the Supplementary).

Based on their similarities with the experiment, the simulations were deemed sufficient to determine various physical parameters (temperature, density, pressure, fluid velocity), which were not determined experimentally. These values were taken at 50 ns, i.e just prior to the beginning of the turbulent phase, to calculate the characteristic dimensionless numbers. However, such calculations could not be performed during the turbulent phase since this phase was not reproduced in simulations.

The simulated values, as well as some derived parameters are presented in Table 1. The viscosity, required for the Reynolds number, is calculated with the multi-material formula established by Clérouin^43^, which can be applied to partially ionised plasmas. A Thomas-Fermi model is used to determine the ionisation of each species. These values are used to estimate the Reynolds number at the beginning of the turbulent phase, which is found to be of the order of 10^7^. This high value, rarely attained in simulations due to numerical viscosity, is consistent with turbulence theory^42^.

**Table 1:** Plasma parameters. Summary of different plasma parameters relevant to the flow. The “simulated parameters” are deduced from simulations. They are taken in the foam near the RTI peak at the proximity of the interface. The “calculated parameters” are determined through the formula (in cgs units, amu is the atomic mass unit) using the simulated parameters. Alternative values for the parameters depending on *L* and *u* can be found in Table 2, where a more conventional calculation method is used.

Simulated Parameters	Formula	MULTI	FLASH
Interface Position (*L*) [μm]	Simulated	388	410
Foam density (*ρ*) [g cm^−3^]	Simulated	0.2	0.22
Temperature (*T*) [eV]	Simulated	1.1	0.8
Pressure (*P*) [kbar]	Simulated	43	26
Fluid velocity (*u*) [km s^−1^]	Simulated	5.7	5.5

Calculated Parameters			
Foam ionisation (*Z*_C_, *Z*_H_, *Z*_O_)	Thomas-Fermi^a^	0.9, 0.4 and 1	0.9, 0.4 and 1
Mean ionisation (*Z*)	(15*Z*_C_ + 12*Z*_H_ + 4*Z*_O_)/31	0.7	0.7
Mean ion mass (*m*_i_) [10^−23^ g]	(15*m*_C_ + 12*m*_H_ + 4*m*_O_)/31	1.37	1.37
Ion density (*n*_i_) [10^22^ cm−^3^]	*ρ*/*m*_i_	1.38	1.60
Viscosity (*ν*) [10^−4^ cm^2^ s^−1^]	Clérouin formula^43^	5	4
Reynolds (Re)	*uL*/*ν*	4 × 10^7^	6 × 10^7^
Euler (*Eu*)	*P*/(*ρu*^2^)	0.7	0.4
Kolmogorov length (*η*) [nm]	*L*/Re^3/4^	0.7	0.6
Electron inertial length [nm]	$5.31\times 1{0}^{5}\cdot {n}_{\,\text{e}\,}^{-0.5}$	52	50
Ion inertial length [μm]	$2.28\times 1{0}^{7}/Z\cdot {({m}_{\text{i}}/(\text{amu}\cdot {n}_{\text{i}}))}^{0.5}$	7.7	7.4

^a^Use of the programme developed by Murillo Group from the Michigan State University. Those programme can be found on github: https://github.com/MurilloGroupMSU/Dense-Plasma-Properties-Database

Alternative approaches can be taken to calculate the Reynolds number at 50 ns. They mainly differ in their definition of the characteristic velocity and length of energy injection. For instance, an upper value of the Reynolds number can be calculated using the actual plasma extension as well as the fluid velocity (see Table 1). In this case, it will be equal to 6 × 10^7^. We can also use the mixing zone of the RTI and its growth velocity, which is actually equivalent to the fluid velocity in the reference frame of the interface (see Table 2). With this more common definition, the Reynolds number drops to 1 × 10^7^. Finally, if we consider the spacing between RTI spikes and the variation of fluid velocity in the lateral direction as a constraint for our system, we find Re ~ 7 × 10^6^ (see last column of Table 2). These choices give roughly the same order of magnitude for Re and all remain consistent with turbulence theories.

**Table 2:** Alternative values of the plasma parameters. This table is an alternative of the value found in Table 1 when using the reference frame of the expanding interface. Since the values presented here depend on the RTI development, only values obtained using the 2D simulations (FLASH) are shown. The last column of the table corresponds to value taken in the transverse direction. *L* is the spacing between spike and *u* the lateral variation of velocity. This values were take from the hydrodynamic simulation of the mono-mode case.

Parameters	MHD bi-mode	MHD mono-mode	Hydro mono-mode	Hydro lateral
Mixing Zone width (*L*) [μm]	120	105	134	65
Characteristic velocity (*u*) [km s^−1^]	1.5	2.1	2.9	4
Reynolds (Re)	5 × 10^6^	6 × 10^6^	1 × 10^7^	7 × 10^6^
Euler (*Eu*)	3.7	1.9	1.4	0.8
Kolmogorov length (*η*) [nm]	1.2	0.9	0.7	0.5

### Spectral analysis

In order to experimentally study the turbulent phase, which appears in late time radiographs, regions of interest (ROI) were defined in which the flow appears to be turbulent (see red squared on Fig. 3). A spectral analysis of each ROI was performed. To this end, the mean radial power spectrum of ROIs was determined (see method), assuming isotropy. While this is a very good approximation during the turbulent phase, this breaks down at early times (see Fig. 2 and the spectral analysis section of the Supplementary). Images of the XFEL beam itself, radiographs of non-driven target, unshocked foam in driven shots, and early time results, were also analysed as references.

The obtained intensity spectrum can be linked to the imaginary part of the optical length. At early times, it relates to a density spectrum. Later in time, in the turbulent phase, the density tends to become homogeneous, and so the intensity spectrum indicates the bromine concentration. Given our experimental conditions, the post-shock flow is subsonic and as a consequence the density fluctuations and the velocity spectrum should be the same^44,45^. In other words, at early times, the density fluctuations (or more precisely the volumetric x-ray attenuation) should behave as a passive scalar, which is transported with the flow. At later times, the bromine concentration is the passive scalar, which is transported. The presence of PC may also alter the obtained spectrum. However, only high spatial frequencies may be affected, since the longest PC wavelength measured for high optical index gradient is of the order of 3 μm (see supplementary information).

**Fig. 4:**
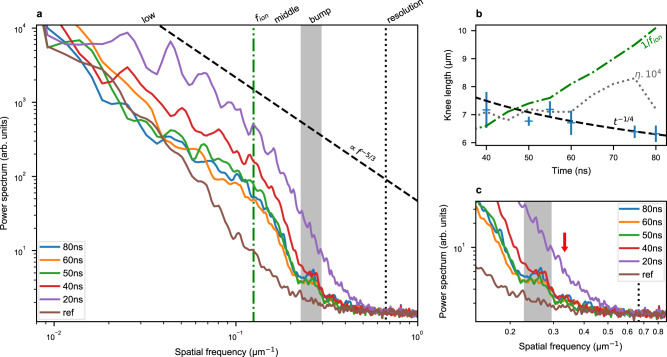
Spatial power spectra of the turbulent zone. Spatial power spectrum (**a**), temporal evolution of the knee position (**b**) and bump morphology (**c**). **a** Typical radial power spectrum of the turbulent plasma obtained, at different times after the laser drive, compared to a reference case, non-shocked foam. The “low” spectrum evolved as a power law consistent with turbulence theory: a fiducial with a −5/3 coefficient is marked by dashes. The middle zone follows a power law with a much higher coefficient (nearly −6.6 in the 60 ns spectrum). The spatial frequency corresponding to the ion inertial range at 50 ns, *f*_i_, is marked by the dash-dot line and is near the inflexion of the spectrum. The bump is highlighted by the grey zone, and is abnormal considering classical turbulence theory. The nearly flat spectrum, for spatial frequency above the bump, corresponds to the resolution limit of our diagnostic. **b** Evolution of the knee position as a function of time. The data points correspond to the mean values of our data set with a standard deviation as error bars. The dashed fiducial corresponds to a *t*^−1/4^ evolution. The dotted line corresponds to the simulated Kolmogorov scale (as calculated in Table 2 for the hydrodynamic mono-mode case) time of 10^4^. The green dash-dotted line to the theoretical ion inertial range. **c** Comparison of the bump morphology at different times. The greyed zone highlights the bump position. The red arrow marks the phase contrast wavelength measured on grids.

Typical spectra can be decomposed into four zones, as shown in Fig. 4. For the lower wave numbers (<2 × 10^−1^ μm^−1^) we can distinguish two spectral distributions. We qualify them as “low” and “middle” spectra, with an inflection at a knee spatial frequency, *f*_k_. At higher wave numbers, a bump appears at late times, during the turbulent phase. For very high spatial frequency, *f*, the spectra become almost flat with some noise. This corresponds to the resolution limit of the radiography (~1.5 μm).

Once the isotropy is established at late times, the “low” spectrum becomes consistent with standard turbulence theory. It scales as a power-law *f* ^*q*^, where *q* increases with time and tends to *q* = −1.75 ± 0.25. This is consistent with the Kolmogorov theory (*q* = −5/3), as well as other turbulence theories: *q* = − 7/4 when turbulence evolves from RTI^46^, *q* = −1.5 from RMI^46^, or when *q* is a summation of harmonics^47^. One should note that the length scale of the energy injection, which as a minimum is of the order of the RT mixing zone^47^, is larger than the inertial range (the range over which the spectrum follow the Kolmogorov power-law), as expected. We can also note the loss of the initial conditions, as the spectrum does not reflect the pre-imposed modulations (15μm and 40 μm ).

The “low” spectrum ends at the knee spatial frequency, which marks the beginning of the “middle” spectrum. The associated spatial length, the knee length ${f}_{\,\text{k}\,}^{-1}$, varies in time (see Fig. 4b) going from 7.2 ± 0.6 μm at 40 ns to 6.3 ± 0.3 μm at 80 ns. As a result, the inertial range (range of the “low” spectrum) grows in time. This is consistent with actual turbulence evolution.

As in the “lower” spectrum case, the “middle” spectrum follows a power-law, but with a much higher exponent, 6.6 ± 1.3. This can be consistent with power spectrum found for wavelengths above the dissipation scale, as those also show an abrupt decrease^48^. This would entail that the knee length, ${f}_{\,\text{k}\,}^{-1} \sim 7\ \mu$m, corresponds to the Kolmogorov length (*η*). However, the obtained Kolmogorov length would be far from the nanometric length predicted by the classical theory for the involved Reynolds number (see Table 1). Another interpretation seems more coherent, the “middle” spectrum may correspond to a plasma turbulent spectrum in its sub-ion range^49^. In this case, the knee-length would either correspond to the ion inertial length or to the ion gyroradius, depending on the plasma conditions. These interpretations will be discussed in details in the “Discussion” section.

The spectrum, determined at early times during the RTI growth (before 40 ns), is not well established and the transition between the two parts is smoother (no clear inflection point). However, the radial projection is not well-adapted to this phase since its 2D spectrum is anisotropic (see Section VI of the Supplementary). Hence, in addition to the observation of the turbulent phase, we have diagnosed the transition between a 2D flow to a 3D stochastic flow. It is interesting to note that this is an observation of a turbulent spectrum at this spatial scale and of such a transition in a laser-plasma experiment. This observation was made possible by the high resolution of our experimental platform.

The main difference between our measured spectrum and any theory is the presence of the bump. It corresponds to a structure with a spatial scale of 3.9 ± 0.1 μm, which is smaller than the knee length. This structure becomes more pronounced at later times (see Fig. 4c) and is absent at early ones. Thus, we can not associate this structure to an effect of the PC. This is because PC effects should be more important at early times, when the gradient of optical indexes is higher. Furthermore, we can also distinguish a smaller bump, whose spatial frequency corresponds to the dimension of the measured PC (~3 μm red arrow). Therefore, the bump seems to be linked to the turbulent flow.

Such an observation is unprecedented and this “bump” structure is not predicted by purely hydrodynamic turbulence theory. It may be the result of energy injection at a given spatial scale, possibly due to non-local energy transport such as magnetic coupling (the eddies increase magnetic fields by the dynamo effect^45^), or it might be linked to the near resolution limit. A full explanation of this feature, however, is beyond the scope of this article.

This experiment represents a milestone in HEDP study, as it is a direct experimental measurement of the turbulent spectrum over a large order of spatial frequency and down to microscopic scale, in a laser-produced plasma experiment. It reveals characteristics (inflexion and bump), which, to our knowledge, have never been experimentally observed in a controlled environment. The numerous features, observed here, are essential for benchmarking models and simulations of plasma turbulence in HED systems. Moreover, with our platform, suitably designed experiments could solve outstanding problems in HEDP either in ICF or in astrophysics, where turbulence is of central importance.

## Discussion

Two particular features of the previously shown spectra are worth discussing since they diverge from the usual turbulence theories. Those are the inflexion between the “low” and “middle” spectra (knee) as well as the “bump”. Despite our lack of definitive explanation for those features, we can provide some substantiated hypotheses.

As previously alluded to during the spectra description, the knee length could correspond to the Kolmogorov length or to an ion characteristic length. For both cases there are arguments promoting and opposing them. A point in common for both arguments is the sudden drop in the power spectrum for spatial frequency above *f*_k_ (“middle” spectrum), although this, in itself, is insufficient. Two others points are worth considering: the actual value of the knee-length compared to its theoretical counterpart, and the temporal evolution of this value.

Starting with the value of the knee length, one can rule out the possibility of it being the Kolmogorov length. Indeed, the knee-length evolves between 7.5 and 6.0 μm this value is far from the usual few nanometres of the Kolmogorov length. The theoretical Kolmogorov length of our system is lower than a nanometre (see Tables 1 and 2) according to the classical turbulence theory (scaling as Re^−3/4^ see Method). Although this theoretical approach relies on ideal hydrodynamics, whose approximations are not perfectly valid in this case, the actual Kolmogorov length should not be three orders of magnitude higher.

In the case of ionic effects, the transition between inertial range and sub-ionic range would either happen at the ion inertial length when the plasma *β* is lower than 1 or at the ion gyroradius when it is higher than 1^49^. According to the values reported in Tables 1 and 2, the ion inertial length seems similar to the measured knee length. However, the *β* parameter of our system should be much higher than 1, given our estimations of the magnetic field generated by Biermann battery effect (see Method and Section IV of the Supplementary). Furthermore, the ion gyroradius of our system should be of the order of a few millimetres, or more, given our low magnetic field. So this explanation does not match well.

The second point to consider is the temporal variation of the knee length. As previously described, this length seems to weakly decrease during the experiment. According to J.R. Ristorcelli^50^, in the case of turbulence evolving from a RTI, the Kolmogorov length evolves as *h*^−1/8^, with *h* the RTI amplitude or mixing zone width. In the most commonly studied case, this amplitude evolves as *t*^2^ during the asymptotic regime of the RTI. This leads to a Kolmogorov length evolution as *t*^−1/4^. Such evolution would agree with our knee-length evolution; however, our mixing does not follow this kind of growth (see Fig. S2 in Supplementary). In our case, we would rather expect an evolution as *t*^−*a*/8^ with 0 < *a* ≲ 1. Furthermore, we should note that in ref. ^50^, the viscosity is taken to be constant as a hypothesis. This does not seem to be the case in our experiment according to our simulations and resulting calculations. Thus, the above-mentioned theoretical evolution might not be perfectly suitable in this experiment. This is supported by our simulation results, from which we predict a quasi-constant Kolmogorov length (see grey dotted curve on Fig. 4b).

According to our simulations, the ion inertial length should grow in time (see green dash-dotted curve on Fig. 4b). So even if its value may correspond to the knee position at ~50 ns, it does not share its dynamic evolution. One might argue that our simulations can no longer be fully trusted after the beginning of the turbulent phase, but the growth of the ion inertial length start before. After considering these arguments, we are therefore still not able to definitively understand the nature of the inflexion point.

As mentioned previously, the bump is an unexpected feature of the spectra, which becomes more pronounced in time. Its characteristic length, ~3.9 ± 0.1 μm, does not correspond to the effect of PC nor to any other diagnostic-based effects. Considering the existing turbulence theories and current experiment and simulations, this bump could call some 2D turbulence spectra to mind^51,52^. For such spectra, a bump appears between the direct cascade, which transports the energy to the smaller spatial scale, and the indirect cascade, which transports the energy to the higher scale (a specificity of 2D turbulence). The boundary between both cascades corresponds to the energy injection scale, which is marked by a bump in the energy spectrum.

Following this comparison, the bump may correspond to a length characteristic of an energy injection in the system. However, the nature of such injection remains a mystery. The main source of energy for the system is the optical laser. Despite its large focal spot (240 μm), which can not be associated with the bump, it possess numerous smaller perturbation in the form of speckles. However, the bump appears only at late times, whereas the laser is shut down after 4.5 ns. So it does not seems that they could be directly linked.

One of the particularities of the observed turbulence is that it happens within a plasma flow, and such flows can be subject to electromagnetic constraints. Initially, there is no pre-imposed magnetic field. The magnetic field produced by the laser and its interaction with the target should disappear with the laser. However magnetic fields can be produced as a result of plasma dynamics. According to our estimation (see method for the details), a magnetic field of ~0.1 G is produced around the interface position due to Biermann battery effects (this is an overestimation of its actual value). Because of its low value, the magnetic pressure is negligible compared to the thermal pressure; i.e. the *β* of the plasma is high. The magnetic Reynolds number of the system is also relatively low (see methods). As a result, the magnetic field diffuses in the plasma. It might still carry energy between spatial scales, but its efficiency should also be low. Another downside of this explanation is understanding the scale of the energy injection. Indeed, we do not explain why a self-generated magnetic field would present such a specific spatial scale.

Another interpretation would be to think of the bump as an artefact of our experiment and diagnostic. We have already ruled-out the possibility of a PC effect, but one could imagine other lengths, which characterise our target, or the materials present (for instance the pusher granularity or the size of the pore in the foam) might be responsible. However, these lengths are already present from the beginning and thus there is no reason for the bump to appears only later in time. However the only sound evidence for this would be obtained by redoing the experiment with a different target design. For instance by using other materials processing techniques, the granularity of the target will change, and the use of different foams density will change the pore size. This will be the subject of an upcoming experiment.

It is worth mentioning that the experimental technique presented here can be applied to other systems unrelated to turbulence. The high spatial and temporal resolution of our diagnostic, its large field of view, as well as the monochromaticity of the XFEL beam, are assets, from which the laser-plasma community will largely benefit. The possibilities are numerous: from studying small details of physical phenomena (subtle morphology of diverse instabilities and the effect of magnetic fields on them, separation of the elastic and plastic wave in a shock) to the measurement of the actual plasma density using the XFEL monochromaticity (possible application for the measurement of equation of state, or heat transport...). The main down-side of this experimental technique is the requirement to couple an XFEL to an optical high power laser and the rarity of such facilities. However, active development is being performed at LCLS, SACLA and Eu-XFEL, where an external magnetic field will also be added.

## Methods

### Target composition

The target used during this experiment is similar to a previously tested target^28^. This is a multi-layer target composed of a 10 μm parylene ablator, a 40 μm modulated brominated plastic (C_8_H_7_Br) pusher and a resorcinol formaldehyde (C_15_H_12_O_4_) foam (100 mg cm^−3^). The experiment was performed in SACLA experimental hutch 5. An optical laser deposits ~20 J on a ~240 μm diameter focal spot, which is smoothed by a hybrid phase plate (HPP), in a ~4.3 ns square pulse giving an intensity of ~1 × 10^13^ W cm^−2^ on target. This leads to the ablation of the first layer, the ablator, and in reaction generates a shock wave inside the solid target. The shock wave, during its propagation, puts into motion the rest of the target, in particular the pusher that expands into the foam and decelerates. This situation is Rayleigh–Taylor unstable.

Most of the pushers were modulated in order to make this experiment reproducible and easy to simulate. Indeed, this allows the control over which spatial modes will be favoured during Rayleigh–Taylor development. These modes will not be created randomly due to thermal noise, material roughness, imperfection in the spatial deposition of the laser energy, or numerical noise, in the case of simulation. Two kinds of modulation were used: a simple sine wave (mono-mode), with 40 μm wavelength and 5 μm amplitude; the sum of two sine waves (bi-mode), the previous one and another with 15 μm wavelength and 5 μm amplitude.

### Imagery technique

The imaging system consists of a collimated x-ray beam and a LiF crystal. When irradiated by x-rays the LiF creates colour centres (CCs) that can be read out using a conventional confocal fluorescent microscope^23^. Here, the XFEL beam consists of a short pulse (less than 10 fs) x-ray burst centred on a 7 keV peak, with a ~30 eV full width at half maximum (FWHM)^35^. It is collimated so the resulting radiography images on the LiF have the same dimension as the imaged object (contact radiography, i.e. magnification of 1). The intrinsic resolution of this system depends on the spacing of the colour centres within the LiF (a few nanometres), the resolution of the confocal microscope used to read out the data (0.26 μm), and the secondary electron avalanche and diffusion of colour centres, that appear in the LiF^37^. This last aspect seems to be the limiting factor in this experiment, as T. Pikuz^38^ suggested the formation of a ~0.6 μm diameter electron cloud in a similar configuration (XFEL, with an energy of 10 keV). In this experiment, the image contrast is ensured initially by the difference of density of the different materials involved (initially the foam is more than ten times lighter than the pusher), then by the difference in the absorption coefficients of those materials (the high Z material, Br, absorbs more x-rays), and by phase-contrast (PC) effects. The PC effects lead to the appearance of structures which were measured to be ≤3 μm, the upper limit corresponding to a static case, i.e. with no laser drive, on a 400 line per inch gold grid. This estimation was obtained through direct measurement of the structure (radiography of the grid, observation of the tip of the RTI peaks), by line-outs, and also by performing our spectral analysis, based on fast Fourier transforms, on those line-outs. These three methods were applied on locations were PC was obvious, such as the limit between the foam and the pusher. The PC structures do not correspond to the measured bump wavelength, all structures spatially larger than 3 μm are due to absorption effects.

### Spectrum calculation

To determine the power spectrum, several steps were required. First, the regions of interest (ROIs) were cropped from the figure; they correspond to regions either completely included in the shocked regions or entirely outside (unperturbed foam) used as reference. The typical size of the ROIs were 520 × 520 pixels with a pixel size of 0.31 μm, although some other dimensions (720 × 720, 1040 × 1040...) were used for checking the method consistency. A fast Fourier transform was applied to the ROI, and the square of its norm taken. This corresponds to the 2D power spectrum. These spectra were projected onto polar coordinates and averaged over the angle to obtain a radial power spectrum (see Fig. 4 for typical results). An average over the radius was also performed to verify the isotropy of the system (see Fig. S6 in the Supplementary). Only the data taken after 30 ns are isotropic. Finally, the method was applied on higher magnification images with a pixel size of 0.155 μm to ensure the absence of artefacts due to the specific configuration of the microscope.

### Simulations of the experiment

As described in the main part of the article, we used some hydrodynamic simulations to obtain most of the physical parameters that characterise our plasma. Two radiative hydrodynamic codes, with laser-matter interaction module, were used: FLASH4 and MULTI.

FLASH4 (version 4.5 in this article), which is developed at the Flash Center (University of Chicago)^24,25^, is a multidimensional hydrodynamic and magneto-hydrodynamic Eulerian code with an adaptive mesh refinement (AMR) scheme. We used it in a 2D Cartesian configuration over an 400 × 800 μm domain for a maximal resolution of ~0.8 μm. This code was used both in hydrodynamic, solving the Euler equations of fluids dynamic, and magneto-hydrodynamic without any external magnetic field modes, both configuration returning similar results (see Table 2).

MULTI^53^ is a 1D Lagrangian code. Thank to both aspects, the resolution of the performed simulations are higher than with FLASH4. However the global morphology of the plasma flow can not be studied (only 1D).

For both types of simulation, the laser intensity is used as an adjustment parameters to reproduce the initial interface velocity. The resulting simulations can be directly compared to our experimental results with good adequacy (see Supplementary Information). However, the turbulent phase was not reproduced. Thus the simulation results are no longer valid after the beginning of the turbulent phase.

### Calculation of Reynolds number

For the Reynolds number calculation (Re = *uL*/*ν*), the characteristic length, *L*, velocity, *u*, and viscosity, *ν*, are needed. The foam viscosity was calculated using the Clérouin formula, which requires the shocked foam temperature (~1 eV), its density (~0.2 g cm^−3^), its composition and ionisation (calculated through a Thomas-Fermi model). To determine these parameters, we used the above-mentioned simulations performed with the FLASH4. We should note that the temperature and density obtained through simulation were similar to the ones given by the equation of state tables for our measured shock velocity. The characteristic length and velocity can be either taken to correspond to^42^: the plasma extension and bulk velocity, the RTI extension and the associated growth velocity, or the inter-spikes extension and the lateral velocity. The exact definition depends on which scale we consider for energy injection. The first possibility would usually be taken in jet like objects (the global morphology of our expanding plasma), whereas the second and third are more usually used for theoretical study of the RTI and resulting turbulence (usually it is associated with periodic boundaries). We should note that the spacing between spikes grows in time as they diverge from one another due to the curvature of the expanding plasma. In the case of bulk flow, the length is taken to be equal to the plasma expansion: ~400 μm at 50 ns. The velocity was taken to be the post-shock fluid velocity in the foam (~7 km s^-1^). The resulting Reynolds number is of the order of 10^7^, which is consistent with turbulence theories (other possibles values are written in Table 2).

Another estimation of the Reynolds number is possible by using some properties of the turbulent spectrum. In the classical theory, which does not apply in our case (our experiment is neither purely hydrodynamic nor incompressible), the Reynolds number is linked to the injection spatial scale, *ℓ*, and to the Kolmogorov scale, *η*, by the formula Re = (*ℓ*/*η*)^4/3^. Since we do not measure *η* in our experiment, we can use this formula to calculate the actual dissipation scale. Here, we obtain a Kolmogorov scale of the order of a nanometre (see Tables 1 and 2, and Section III of the Supplementary).

### Magnetic field

The calculation of the magnetic field present in the plasma and related parameters is necessary to evaluate its effect on the spectra. In this experiment, no external magnetic fields were directly applied on the target. The sole magnetic fields present are either produced by laser-matter interaction (but they disappear after the laser pulse), or by the plasma evolution. Here we only consider magnetic fields produced by the Biermann battery effect. For this, we calculate the cross product of the gradient of electronic temperature and electronic density in the bubble at 50 ns using the simulations results. Using the Biermann battery formula, we obtain a temporal variation of magnetic field of: ∂_t_*B* = ~ 2 mG ns^−1^. Supposing that: this value is constant and that the magnetic field is advected with the plasma (stays in the bubble), we calculate a magnetic field of ~0.1 G after 50 ns. This is an overestimation of the real magnetic field, as there is magnetic diffusion. Using this value and the Spitzer formula to calculate the resistivity, we find a magnetic Reynolds number lower than 5 × 10^−2^ (no magnetic advection), a ion gyroragius of the order of 6.5 mm or more for a lower magnetic field, and a plasma beta much greater than 1. Further details and discussion can be found in the Supplementary Information.

## Supplementary information

Supplementary Information

## Data Availability

Raw data generated during the experiment (x-ray radiographs) are available from the corresponding author on reasonable request.
