# Supplementary material for: Micron-scale phenomena observed in a turbulent laser-produced plasma
Source: Nat Commun. 2021 May 11;12:2679. doi: 10.1038/s41467-021-22891-w (PMC8113596; doi:10.1038/s41467-021-22891-w)
Supplement: Supplementary file 1 — Supplementary Information [file 41467_2021_22891_MOESM1_ESM.pdf]

Supplementary information
Micron-scale phenomena observed in a turbulent laser-produced plasma

G. Rigon[a],[1] B. Albertazzi,[1] T. Pikuz,[2,3] P. Mabey,[1] V. Bouffetier,[4] N. Ozaki,[5,6] T. Vinci,[1] F. Barbato,[4] E. Falize,[7] Y. Inubushi,[8,9] N. Kamimura,[5] K. Katagiri,[5] S. Makarov,[3,10] M.J.-E. Manuel,[11] K. Miyanishi,[9] S. Pikuz,[3,12] O. Poujade,[7,13] K. Sueda,[9] T. Togashi,[8,9] Y. Umeda,[5,14] M. Yabashi,[8,9] T. Yabuuchi,[8,9] G. Gregori,[15] R. Kodama,[5] A. Casner,[4] and M. Koenig[1,5]

[1] *LULI, CNRS, CEA, Ecole Polytechnique, UPMC, Univ Paris 06: Sorbonne Universites, Institut Polytechnique de Paris, F-91128 Palaiseau cedex, France*
[2] *Institute for Open and Transdisciplinary Research Initiative, Osaka University, Osaka, 565-0871, Japan*
[3] *Joint Institute for High Temperature RAS, Moscow, 125412, Russia*
[4] *Université de Bordeaux-CNRS-CEA, CELIA, UMR 5107, F-33405 Talence, France*
[5] *Graduate School of Engineering, Osaka University, Osaka, 565-0871, Japan*
[6] *Institute of Laser Engineering, Osaka University, Suita, Osaka 565-0871, Japan*
[7] *CEA-DAM, DIF, F-91297 Arpajon, France*
[8] *Japan Synchrotron Radiation Research Institute, 1-1-1 Kouto Sayo-cho, Sayo-gun, Hyogo 679-5198, Japan*
[9] *RIKEN SPring-8 Center, 1-1-1 Kouto Sayo-cho, Sayo-gun, Hyogo 679-5148, Japan*
[10] *Department of Physics of accelerators and radiation medicine, Faculty of Physics, Lomonosov Moscow State University, Moscow, 119991, Russia*
[11] *General Atomics, Inertial Fusion Technologies, 3550 General Atomics Way, San Diego, CA 92121, USA*
[12] *National Research Nuclear University "MEPhi", Moscow, 115409, RUSSIA*
[13] *Université Paris-Saclay, CEA, LMCE, F-91680, Bruyères-le-Châtel, France*
[14] *Institute for Planetary Materials, Okayama University, Misasa, Tottori 682-0193, Japan*
[15] *Department of Physics, University of Oxford, Oxford, UK*

CONTENTS

I. Simulation Parameters 2

II. RTI growth and comparison to simulation 2

III. Flow characteristics 4

IV. Magnetic field and related parameters 4

V. Resolution and phase contrast 5

VI. Spectral analysis 6

References 9

[a] gabriel.rigon@ens-lyon.org

I. SIMULATION PARAMETERS

In this experiment, one main diagnostic (x-ray radiography) was used to study the target interface morphology and dynamics. To access other plasma parameters, such as its temperature, density or pressure, numerical simulations have been performed to reproduce the experimental results. To this end, two hydrodynamic radiative codes were used: FLASH4 and MULTI [1–3].

FLASH4 is a multi-dimensional code developed by the university of Chicago. As a minimum of two spatial dimensions (2D) is required to study the overall morphology of the interface between the pusher and the foam, and thus of the Rayleigh-Taylor instability (RTI) growth, FLASH is well adapted. We use the 4.5 version of FLASH4 in a 2D Cartesian configuration. We work in a hydrodynamic context with two temperatures. An unsplit magneto-hydrodynamic solver was also used but in a context without magnetic fields, its results are close to the hydrodynamic one. The domain was taken to be 400x800 µm, with an adaptive mesh refinement going up to the 6 order allowing for a maximum of 524 288 cells and an effective spatial resolution of ∼0.8 µm. We employed the same equation of state (EoS) for each target layer (ablator, pusher and foam), namely a polystyrene EoS calculated with IONMIX. The radiation is treated using the diffusion approximation with 40 radiation groups. All materials used its own PROPACEOS opacity table [4]. Finally the laser has a 100 µm radius Gaussian focal spot and a square pulse with Gaussian rise and drop (0.15 ns) for a plateau of 4 ns. It delivers an energy of ∼13 J, resulting in an intensity of $1 \times 10^{13}\,\mathrm{W\,cm^{-2}}$.

MULTI is a mono-dimensional Lagrangian code. Therefore, it is not adapted to study the global morphology of the plasma, but is helpful to follow the interface position as a function of time. Furthermore its Lagrangian and 1D nature allow a better resolution in the solid target and the code was calibrated with many experimental results at LULI2000 [5, 6]. Its primary objective is to cross-check the results given by FLASH4. The initial set-up of the simulation consists of 650 µm of material with a total of 1700 cells. A laser delivers ∼9 J in 4 ns, resulting in an intensity of $7 \times 10^{13}\,\mathrm{W\,cm^{-2}}$. Here a scaling factor previously ascertained is used. Here, SESAME tabulated EoS for each material have been used (SESAME7770 for the parylene ablator and SESAME7592 for the polystyrene foam and also for the pusher). In principle SESAME EoS are more reliable than IONMIX, since they were experimentally validated [7], thus the importance of cross-checking.

One should note that the laser intensity used in the simulations does not exactly correspond to the one in the experiment. This is to compensate for the differences between simulation and reality: simulations are not 3D, the laser-matter interaction is not perfectly reproduced, in the experiment the intensity is measured before the target and is thus overestimated. We use the position of our expanding interface to calibrate the laser in our simulations (or use calibration obtained by such means in previous experiments [8]).

II. RTI GROWTH AND COMPARISON TO SIMULATION

In the first part of the experiment (up to 50 ns), the interface between pusher and foam is detectable. As a consequence, one can follow the growth of the RTI. The results, obtained in this phase, already hold a certain amount of scientific interest as mentioned in the article. The high resolution of the diagnostic allows the observation of morphological details, which could not be accessed otherwise. In figure S1, one can see the comparison between our experimental results (A) and a synthetic radiograph (B) obtained from the post-processing of our FLASH simulations. These results correspond to radiographs taken 50 ns after the laser shot in the mono-mode case. Both radiographs (experimental and simulated) present the same morphology with a curved interface and a well developed RTI. Furthermore, as highlighted by the red square and the zoom (C), even more precise details are reproduced in the simulation. Indeed, even the morphology of the head of the RTI spikes matches correctly. These striking similarities show that our simulations correctly reproduce the experiment, and therefore the simulated plasma parameters can be used with confidence.

The main difference between these two radiographs is the blurry aspect present in the experiment but not in the simulations. This might be due to the limitation of our simulations to only two spatial dimensions. As a consequence, our synthetic radiograph does not take into account off-plane perturbations, which contribute significantly to blurring the final image. Furthermore, the plasma flow in our simulation does not become turbulent. This is partially due to the 2D aspect involved in our simulations as well as the numerical viscosity which limits the Reynolds number to non-turbulent values. As can be seen in the experimental radiograph in figure S1, the first effects of turbulence (blurring) can be seen at 50 ns in the mono-mode case, but they are still weak enough for the RTI to still be observed. Thus, this is the last experimental-measured time where the simulation can give us relevant results.

In order to ensure the relevance of our simulations, we also investigated, besides the morphology of the interface, the dynamics of the position of the interface and RTI growth. To this end, the distance between the target rear side (pusher) and the position of the central RTI spike or the extremity of the expanding zone have been measured. This distance is referred to as the interface position. We also determine the distance between the extremity of the

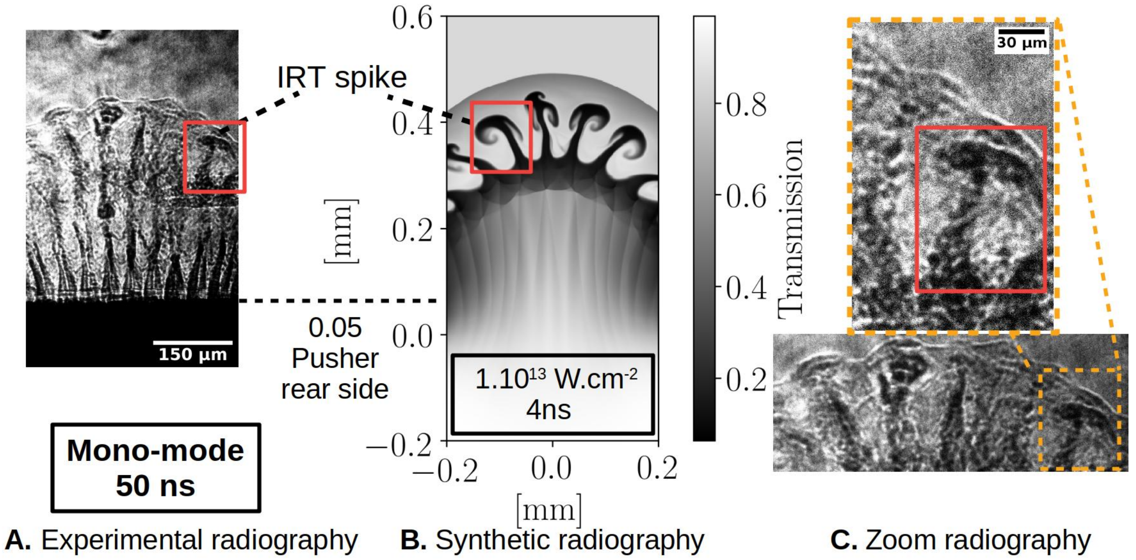

FIG. S 1. Experimental (A) and synthetic (B) radiographs taken 50 ns after laser shot. Both radiographs show a similar overall morphology of the interface, they are align according to the rear face of the target (50 µm thickness). The main difference is the presence of a blurring on the experimental radiograph, which is absent for the simulated one. A zoom of the experimental radiograph (C) illustrates the high resolution of the diagnostic and highlights the morphology of the extremity of the RTI spike.

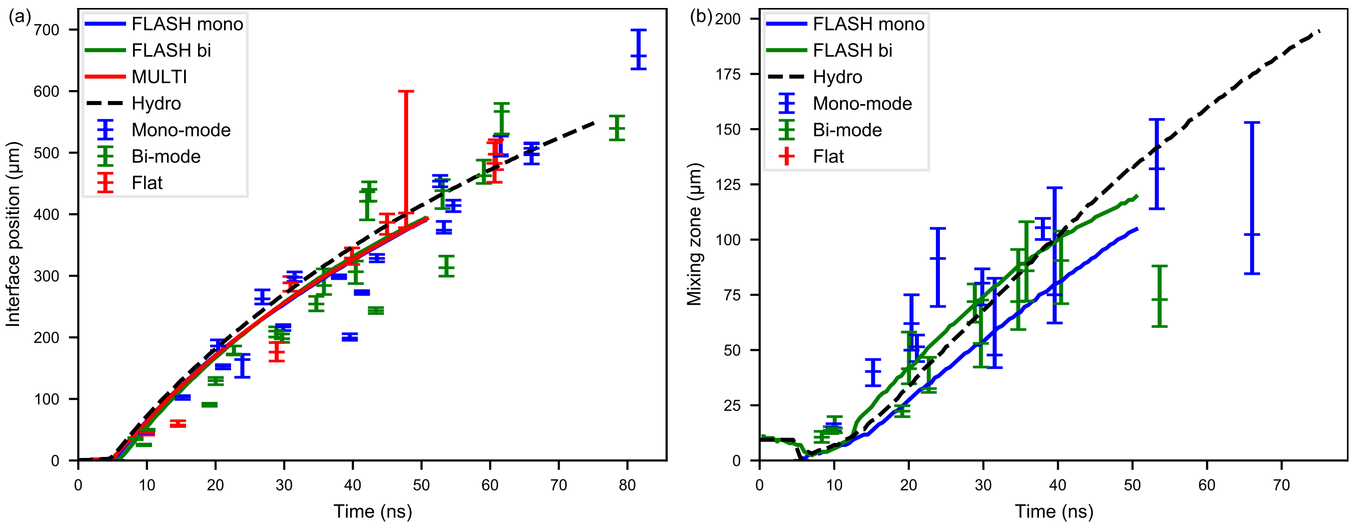

FIG. S 2. Temporal evolution of the interface position (A) and the mixing zone width (B). Each data point correspond to an independent target and laser shot. The associated error bars correspond to measure uncertainties (intensity gradient). The curve corresponds to the simulation results. Since MULTI is a mono-dimensional code, there is no mixing zone width (no RTI development). Three kind of FLASH simulations are presented here: the one named "FLASH mono or bi" where made using an unsplit MHD solver, whereas the one named "Hydro" uses a unsplit hydrodynamic solver in a mono-mode configuration. "Mono" and "bi" refer to the initial modulation of the targets.

central spike and the extremity of the central bubble. This distance, revealing the RTI growth, is referred to as the width of the mixing zone. Figure S2 displays the temporal evolution of these two distances as experimentally measured by the radiography (data point) and as simulated (curve). As expected, there is no obvious difference between the mono-mode and the bi-mode type targets. The interface dynamic´s are identical in both cases, since the impulsion transmitted to the pusher and its deceleration in the foam should be the same (same laser parameters, target composition and thickness and foam density). Observing a similar mixing zone growth is more surprising since the wavelength of the interface modulation should affect the RTI growth. According to our simulations, a difference exists in the mixing zone width between both cases and is hidden in the experimental data due to the error bars. This specific point has already been raised for another RTI laser experiment in [8].

Figure S2 also shows that the simulations produce results that are consistent with the experiment (MULTI for the interface position, FLASH for the interface position and RTI growth). Consequently these simulations are relevant to the description of the experiment up to 50 ns in the mono-mode case and they can be used to determine physical parameters of interest.

III. FLOW CHARACTERISTICS

From the simulations previously described, different physical parameters of the plasma flow can be determined. Those physical parameters, as well as derived values and dimensionless numbers are presented in table I. In the 2D FLASH4 simulations, the most of the parameters were taken in the foam, along the RTI spike axis, near the interface between foam and pusher. More precisely, they correspond to the plateau value which is ahead of the jump due to the interface. The composition of the foam (resorcinol formaldehyde, $C_{15}H_{12}O_4$) is taken into account for the calculation of the ionisation and all that values that depend on it (viscosity, Reynolds number etc.).

The ion inertial length, being a species dependant physical parameter, was calculated for fictitious ions, whose ionisation, density and viscosity correspond to the mean of the values of the real ions in the plasma.

Another approach, which is a better match to the one used in turbulence studies, would be to work in the reference of the interface. In this approach the velocity would correspond to the velocity of the expanding RTI bubbles (time derivative of the mixing zone) and the characteristic length would be taken to be the mixing zone width. In this case, MULTI simulations can not be used to calculate characteristic parameters of the flow since they are 1D (no RTI development). Our FLASH simulations give results, which vary only slightly from one another. The table II summarises the parameters which are different.

IV. MAGNETIC FIELD AND RELATED PARAMETERS

Some of the hypotheses presented in the main body of the article suppose the presence of a magnetic field and its subsequent influence on the energy spectrum. Such a magnetic field was not experimentally measure nor simulated, as the code simulations did not include source terms for the magnetic field. Considering the nature of our experiment, namely a laser produce plasma, two main sources of magnetic field may exist: one linked to laser-matter interaction, the other one to the plasma evolution.

The magnetic field produced by the interaction of the laser with the solid target can reach high values. However, such a field is localised spatially and temporally and will quickly disappear after the laser is shut down. Since the laser lasts only for ∼4.5 ns, there should not be any magnetic field remaining at the beginning of the turbulent phase (around 50 ns).

A second source of magnetic fields is directly linked to the plasma evolution. This source term can be quite complex as it associates diverse contributions. We focused only on the Biermann battery effect here. This effect appears when there is a misalignment between the gradient of electronic temperature and electronic density. It can be expressed as:

$$\partial_{\mathrm{t}}\mathbf{B} = \frac{k_{\mathrm{B}}}{e n_{\mathrm{e}}} \nabla T_{\mathrm{e}} \times \nabla n_{\mathrm{e}} \tag{1}$$

with $\mathbf{B}$ the magnetic field, n_{e} the electron density, T_{e} the electron temperature, k_{B} the Boltzmann constant, e the electron charge, ∂_{t} the partial derivative with respect to time and ∇ the gradient operator.

This formula, applied to the results of our hydrodynamic simulation at $t =$50 ns, gives a magnetic field growth on the order of 20 mG ns^{-1}. This value was estimated in foam, in the bubble. It is obvious that this value might change as a function of time. Thereby, both gradients should be more important at early times and tend to zero when the plasma become uniform at late times. Furthermore, both gradients might not always be misaligned (cross product becoming null) and the magnetic field my not be added at each time steps. For practical reason, we consider this growth to be constant, which leads to a magnetic field of ∼0.1 G at 50 ns. This estimation implies that the field is advected with the fluid, which is not the case in our system.

The advection of magnetic field is unlikely considering the parameters of our plasma and associated magnetic Reynolds number. This number, which compare field advection to diffusion can be expressed as $\mathrm{Re_m} = 4\pi uL/c^2\eta_{\mathrm{r}}$, with η_{r} as the plasma resistivity. By using the Spitzer formula for the resistivity: $\eta_{\mathrm{r}} = 1.15 \times 10^{-14} Z \ln \Lambda T^{-3/2}$ (Z is the plasma ionisation, $\ln \Lambda$ the Coulomb logarithm), we obtain $\eta_{\mathrm{r}} \sim 7 \times 10^{-15}$ sec and $\mathrm{Re_m}$ between 4×10^{-3} and 5×10^{-2} depending on the velocity and length used. Given a value of $\mathrm{Re_m}$ much lower than 1, magnetic field diffusion dominates over advection and hence the latter can be ignored here.

As a consequence of the magnetic field diffusion, our previous estimation of magnetic field creation by Biermann battery effect is an over-estimation of the actual field existing in the experiment. Furthermore since the diffusion

dominates, there is nearly no further amplification of the magnetic field by dynamo effect. Indeed the magnetic field lines do not follow the turbulent fluid motion. Thus they are not stretched and folded by it. For the sake of the following study, we will keep the value of ∼0.1 G at 50 ns, but we will remember that this value is overestimate.

The sub-ionic range of the turbulence spectrum can be linked to the ion inertial length (calculated in the hydrodynamic approximation) or to the ion gyroradius, depending on the plasma β parameter (the last two values rely on the existence of a magnetic field). The ion gyroradius can be expressed as:

$$1.02 \times 10^2 \left(\frac{m_\mathrm{i}}{amu}\right)^{0.5} \frac{T_\mathrm{i}^{0.5}}{ZB} \tag{2}$$

with m_i the ion mass, amu the atomic mass unit, T_i the ionic temperature (in eV), Z the mean ionisation and B the magnetic field. Given the value at 50 ns in table I, and the previously calculate magnetic field, we find a ion gyroradius on the order of 6.5 mm (it is larger in reality as the magnetic field is lower). This value is larger than the extent of our experimental plasma and hence we can conclude that this effect can be neglected here.

The plasma β can be written as $4.03 \times 10^{-11} n_\mathrm{i} T_\mathrm{i}/B^2$. This gives us a β on the order of 10^{13} (or even higher with lower magnetic field). This high value shows that the magnetic pressure is negligible compare to the thermal pressure and thus the magnetic field does not impact the plasma dynamics. However, it can still contribute by injecting energy.

V. RESOLUTION AND PHASE CONTRAST

As highlighted in the main body of the article, the diagnostic used in this experiment presents a high spatial resolution, which allows the observation of details of laser plasma flows. This diagnostic made use of the XFEL beam at SACLA as well as a crystal of lithium fluorine (LiF). The XFEL beam is collimated, with a divergence angle of ∼2 µrad. The LiF crystal was placed ∼10 cm after the target, which gives an optimal resolution of 0.2 µm considering only the geometry of the experiment. As explained in the main body of the article, this resolution is worsened by the electronic cascade that take place inside the LiF crystal. According to [9], this cascade leads to a ∼0.6 µm deterioration of the resolution. Other aspects have also been taken into account when considering the actual resolution of our diagnostic: the intrinsic spatial resolution of the LiF (on the order of a nanometer), the spatial resolution of the reading system (0.31 µm with the confocal microscope and lens used here), the temporal resolution of the diagnostic (∼8 fs leading to a negligible blurring considering the plasma velocity). Finally, the measured resolution is on the order of 1 µm. Since the XFEL beam is collimated the resulting radiograph has no magnification, and the field of view depends on the smallest element between the LiF crystal and the XFEL beam. In our case the field of view is limited by the XFEL beam diameter (∼1 mm).

Figure S3 shows radiographs of static objects to illustrate how our diagnostic can give details with high spatial resolution. In figure S3A, a radiograph of a non-driven mono-mode target is displayed. The diagnostic presents a millimetric field of view in accordance with the XFEL beam diameter. The zoom on the radiograph shows the high resolution of the diagnostic, thus the modulation (40 µm wavelength for a peak-to-valley of 4 µm) can clearly be seen.

In figure S3B, a radiograph of a gold grid 400 lines per inch and an associated line-out are displayed. As can be seen on the line out, the observed signal does not display sharp edges, when the line-out crosses over a bar of the grid, as would naively be expected. The signal presents dampened modulations on each side of the interface between grid and void. These modulations are due to a propagation base phase contrast process. This process is due to the auto-interference of the x-ray beam, which propagates after going through the object. It can be observed because of the high spatial and temporal coherence of the XFEL beam as well as the high resolution of the LiF crystal used as a detector. This phenomenon depends on the gradient of index of refraction, which is especially important in this case considering the presence of gold (at solid density) and void. As highlighted on this line-out, the first phase contrast based modulation has a wavelength of 3 µm. This modulation, which has the largest wavelength and the greatest amplitude, is the one marked as PCI on the spectrum (figure 4). This value does not correspond to the bump.

The phase contrast might also impact the resolution. As can be seen on the line-out, the width of the transition between the gold and void is greater than 1.5 µm. This value corresponds therefore to our resolution limit and is marked as such on the spectrum (figure 4).

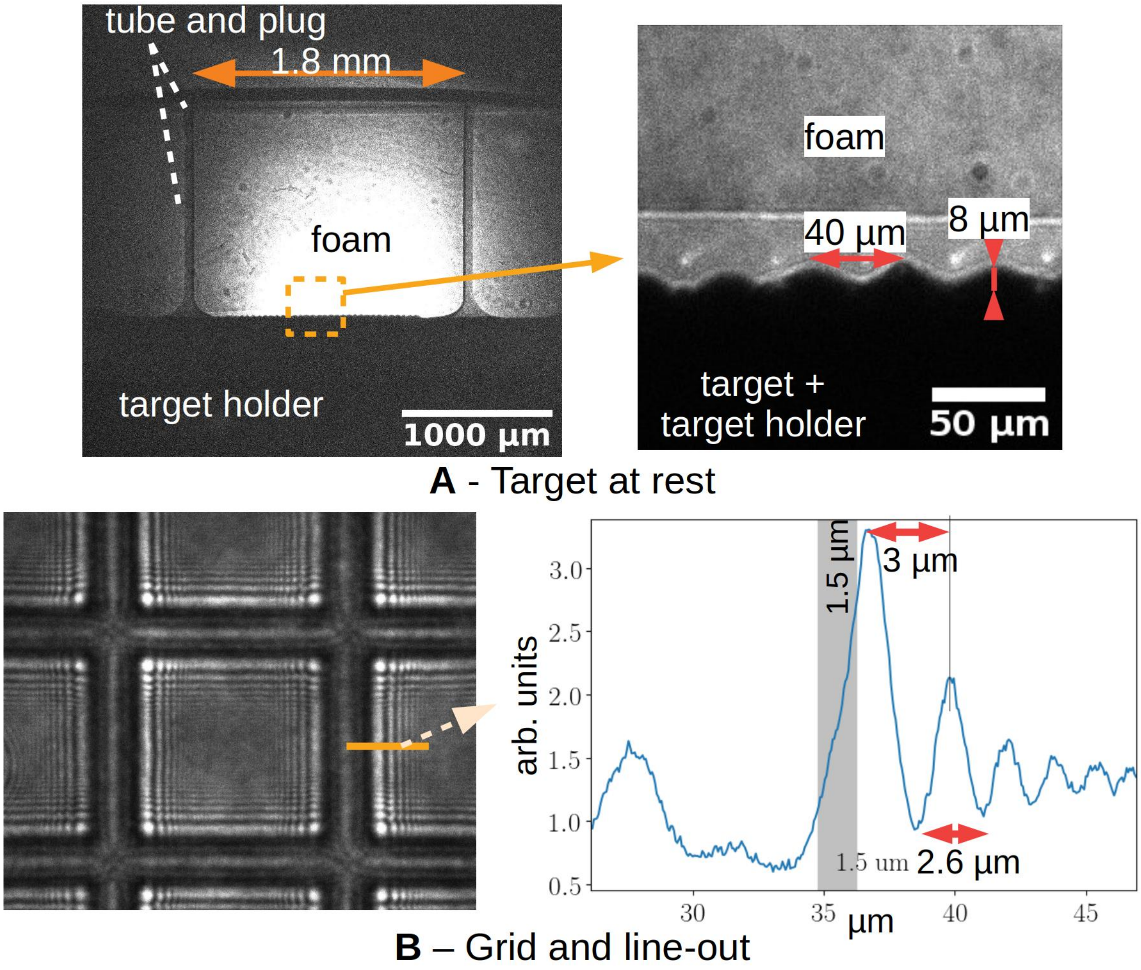

FIG. S 3. X-ray radiograph of static objects. A: Radiograph of a mono-mode target and zoom. The field of view as well as the resolution of the diagnostic are showcase on this radiograph. B: Radiograph of a gold grid (400 lines per inch) and line-out. On the line-out various characteristic lengths are highlighted.

VI. SPECTRAL ANALYSIS

As described in the method part of the main body of the article, the spectral analysis consists of 5 steps illustrated by the figure S4. First, the choice of a square shape region of interest (ROI), second the calculation of the 2D fast Fourier transform, third the calculation of the square of its norm, then its mapping to polar coordinates, finally its average over the angle coordinate to obtain a radial spectrum. The last step consists of a re-normalisation of the spectrum to compensate for a variation of intensity. Since this re-normalisation is just a multiplication of the values of the spectrum by a constant factor, it only corresponds to a vertical translation of the curve (on the log-log diagram). This has no consequences on the study we perform, except for the readability of the diagram.

As can be inferred from this method, the first step is crucial and subjective. Indeed, the choice of the ROI and the choice of its dimension may change the final results. We use square ROI to have the same step size in both direction in the spatial frequency domain. This constraint is not necessary since when mapping to polar coordinates, we linearly interpolate the value for the new grid from the previous coordinates. As described in the method part, we mainly use ROI with a dimension of 520x520 pixels. This dimension is kept constant across our analysis in order to be consistent over different shots, this dimension being the larger square ROI usable at short time (when the plasma did not yet expand appreciably). Other sizes of ROI can be used at later times. However, these do not modify significantly the resulting spectrum. For example, figure S5 shows the spectrum obtained for two sizes of ROI. As can be seen, the resulting spectra possess the same morphology (slope, inflexion point, bump...), indicating that our choice for the size of ROI is unimportant.

Figure 3 shows the different positions of the ROI, which were taken to be entirely in the expanding plasma, so only

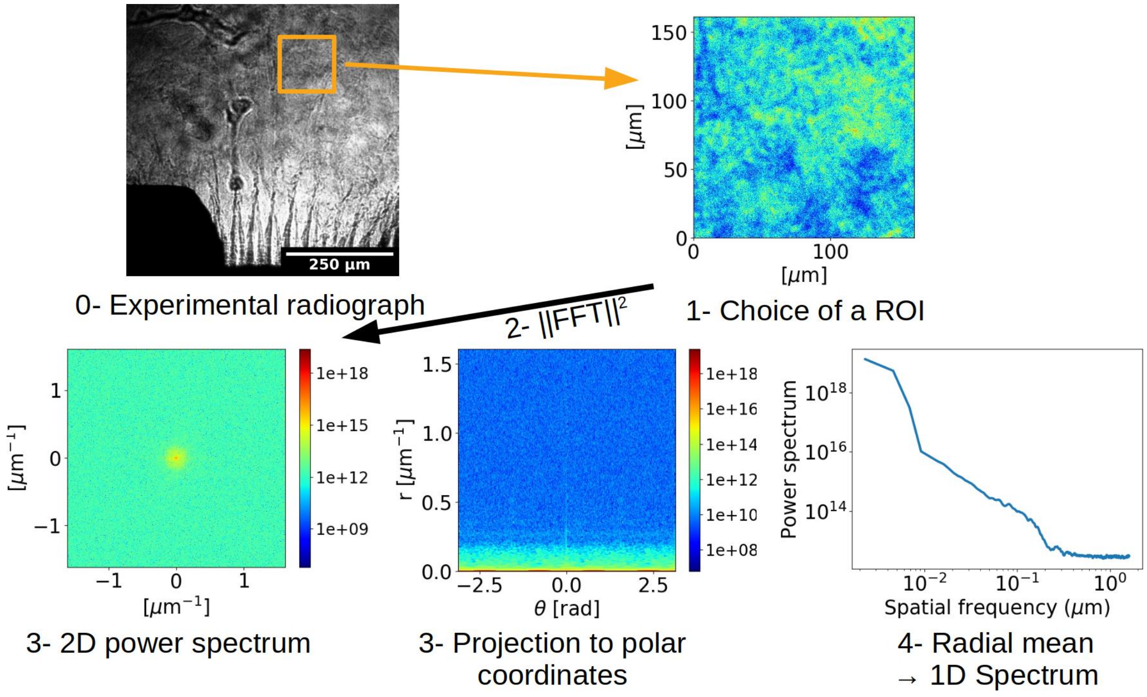

FIG. S 4. Illustration of the steps of the method used for the spatial spectrum calculation. Here a radiograph of a bi-mode target taken 80 ns after laser shot is used as an example.

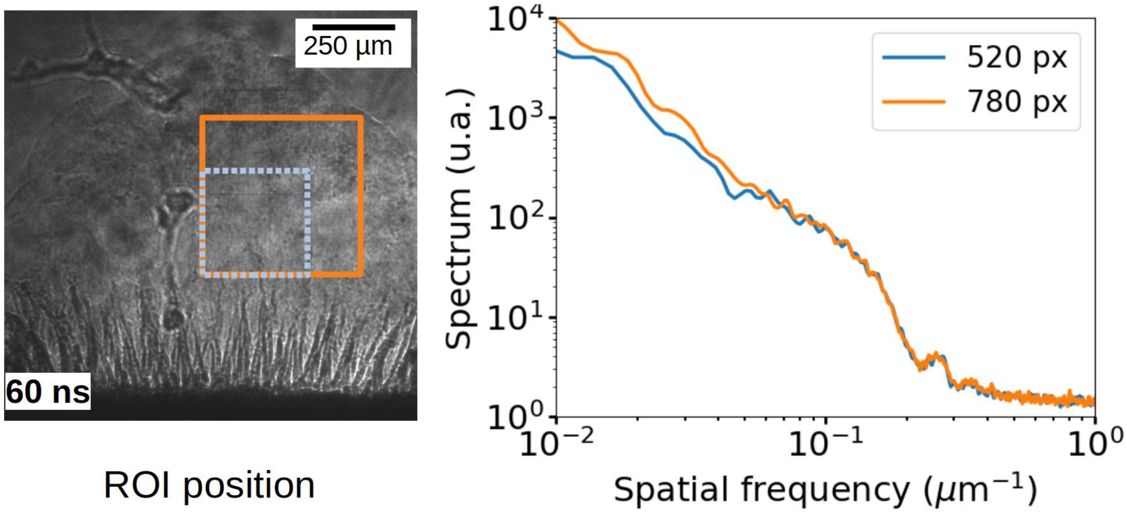

FIG. S 5. Effect of the size of the ROI on the calculated spectrum. On the experimental radiograph taken 60 ns after laser shot two square zones of different size are delimited by a colored line. The spatial power spectrum associated with each line is shown on the graph. There is no important difference between both spectra.

the spectrum of the perturbed part is taken. Furthermore each ROI does not overlap with the XFEL defects, which appears as globular features on the radiographs. Such defects are not due to the plasma but rather the x-ray beam and so should not be taken into account in our study of the turbulent phase.

As can be inferred from the method used to calculate the 1D spectrum, the average performed over the angle coordinate in order to obtain a radial spectrum is valid if and only if the 2D spectrum is isotropic. Figure S6 shows the 2D spectrum obtained when working with the ROI defined in figure 3. As can be seen in this figure, the late time spectra are overall isotropic. Only the spectrum obtained at 20 ns is obviously anisotropic. To estimate the anisotropy of a spectrum, we perform a mean over all radius to obtain a 1D angular spectrum. The standard deviation divided by the mean value of this angular spectrum is a good indication of the degree of anisotropy of the 2D spectrum (0

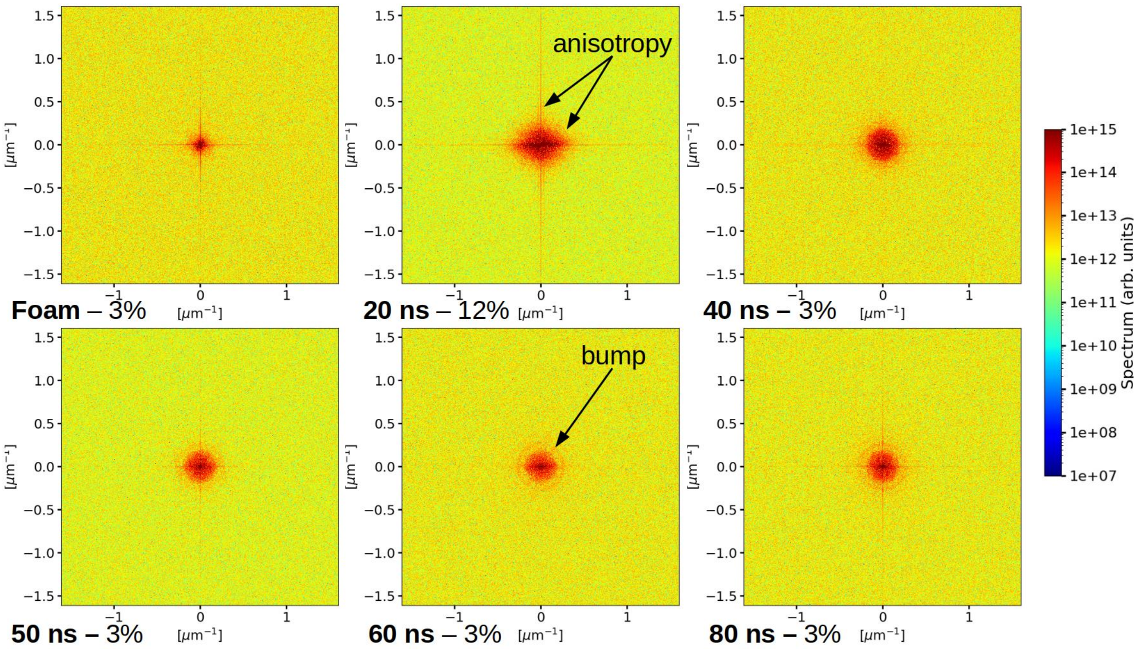

FIG. S 6. 2D power spectrum and associated anisotropy. The 2D spectra, displayed here, correspond to the power spectra, before the projection to the polar coordinates, obtained from the ROIs taken from the figure 3. To each spectrum the associated anisotropy is written in percent.

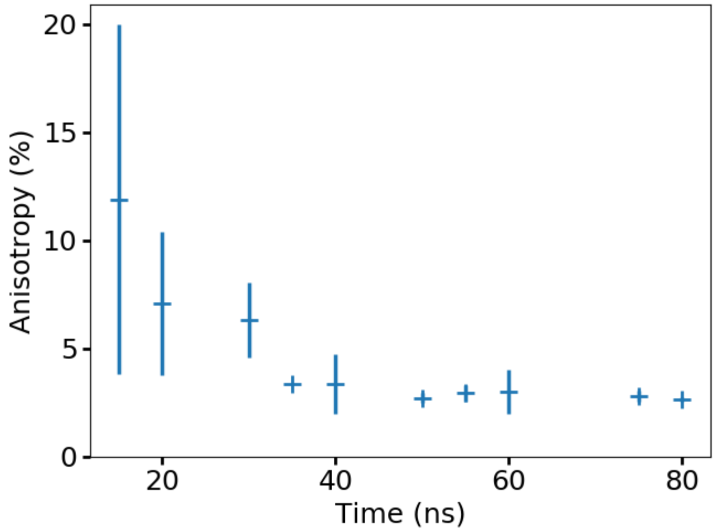

FIG. S 7. Variation of the anisotropic degree as a function of time. The error bars correspond to the standard deviation of our dataset.

being a perfectly isotropic spectrum). In figure S7, the anisotropy degree is given in percentage for each spectrum. As mentioned previously, only the late time spectra are isotropic. The spectra obtained before 30 ns are anisotropic. Figure S7 shows the anisotropy degree as calculated by the previous method over all data (mono-mode and bi-mode targets) for ROI taken at different positions. The error bars correspond to the standard deviation of the data for each time. As observed in figure S7 the plasma flow and the obtained radiography tends to become isotropic.

[1] Fryxell, B. et al. FLASH: an adaptive mesh hydrodynamics code for modeling astrophysical thermonuclear flashes. *ApJS* **131**, 273–334 (2000)

[2] Dubey, A. et al. A survey of high level frameworks in block-structured adaptive mesh refinement packages. *Journal of Parallel and Distributed Computing* **74**, 3217–3227 (2014)

[3] Ramis, R. and Schmalz, R. and Meyer-Ter-Vehn, J. MULTI - A computer code for one-dimensional multigroup radiation hydrodynamics. *Computer Physics Communications* **49**, 475–505 (1998)

[4] MacFarlane, J.J. and Golovkin, I.E. and Woodruff, P.R. HELIOS-CR – A 1-D radiation-magnetohydrodynamics code with inline atomic kinetics modeling. *Journal of Quantitative Spectroscopy and Radiative Transfer* **99**, 381–397 (2006)

[5] Benuzzi, A. et al. Dynamics of laser produced shocks in foam–solid targets. *Physics of Plasmas* **5**, 2827–2829 (1998)

[6] Vinci, T. et al. Temperature and electron density measurements on laser driven radiative shocks. *Physics of Plasmas* **13**, 010702 (2006)

[7] Barrios, M.A. et al. High-precision measurements of the equation of state of hydrocarbons at 1–10 Mbar using laser-driven shock waves. *Physics of Plasmas* **17**, 056307 (2010)

[8] Rigon, G. et al. Rayleigh-Taylor instability experiments on the LULI2000 laser in scaled conditions for young supernova remnants. *PRE* **100**, 021201 (2019)

[9] Pikuz, T. et al. 3D visualization of XFEL beam focusing properties using LiF crystal X-ray detector. *Scientific Reports* **5**, 17713 EP (2015)
